# Psychosocial correlates of free Mpox vaccination intention among men who have sex with men in China: model construction and validation

**DOI:** 10.7189/jogh.15.04070

**Published:** 2025-04-02

**Authors:** Yinghuan Zhang, Meihui Zhang, Yuxuan Wang, Chenrui Li, Huifang Xu, Gang Xu, Jiechen Zhang, Ying Wang, Fan Hu, Yong Cai

**Affiliations:** 1 Public Health Department, Tongren Hospital, Shanghai Jiao Tong University School of Medicine, Shanghai, China; 2 School of Public Health, Shanghai Jiao Tong University, Shanghai, China; 3 Dermatology Department, Tongren Hospital, Shanghai Jiao Tong University School of Medicine, Shanghai, China

## Abstract

**Background:**

The outbreak of Mpox in China has rendered the promotion of effective preventive measures among susceptible populations particularly crucial. We aimed to explore the correlates and develop a model for Mpox vaccination intention.

**Methods:**

We distributed a questionnaire to a sample of 2403 men who have sex with men to investigate whether they would get a Mpox vaccine. The participants were randomly split into a training set and a testing set in a ratio of 3:1. We screened relevant variables by the least absolute shrinkage and selection operator (LASSO) regression analysis and included them into a Mpox vaccination intention model, which used a multivariate logistic regression analysis and presented the findings as a nomogram. We used the receiver operating characteristic curve, calibration curve, Kolmogorov-Smirnov test, lift test, and population stability index to test the validity and stability of the model.

**Results:**

Of the 2403 participants in our sample, 87.1% intended to get an Mpox vaccine. Five of the thirty-one screened variables, *i.e.* Mpox knowledge, social support, vaccination internal rewards, vaccination external rewards, and vaccination response efficacy, were included in the vaccination intention model. The model demonstrated strong risk differentiation (Kolmogorov-Smirnov value = 0.46), moderate predictive power (training area under the curve = 0.7709), and good calibration fit, indicating robust performance.

**Conclusions:**

Our proposed model has a good performance and is highly stable, while our findings suggest that governments should design targeted public health strategies, integrating social engagement and leveraging peer and community education to promote Mpox vaccination.

Mpox, previously referred to as monkeypox, is a zoonotic virus that has predominantly been documented in countries across Central and West Africa over the past four decades [1–3]. In May 2022, a significant outbreak of Mpox was detected in numerous countries that had not previously experienced endemic transmission of the virus [4]. As of March 2024, there have been 95 226 laboratory-confirmed cases of Mpox across 117 countries [5]. On 14 August 2024, Director-General of the World Health Organization (WHO), Dr Tedros Adhanom Ghebreyesus, has determined that the upsurge of Mpox in the Democratic Republic of the Congo and a growing number of countries in Africa constitutes a public health emergency of international concern [6], which brought Mpox back into the spotlight once again. The current Mpox outbreak appears to mainly involve populations of gay, bisexual, or other men who have sex with men (MSM), with transmission primarily occurring through sexual contact [7,8]. This Mpox outbreak also affected China, as the country has reported 2034 cases and ranks among the 10 most affected countries as of March 2024 [5]. This makes the implementation of public health interventions to prevent the spread of Mpox highly significant.

Vaccination is one of the most cost-effective preventive measures for controlling infectious diseases. As of yet, two main vaccines (ACAM2000 and JYNNEOS) have been applied for Mpox prevention [9]. Though these vaccines are not currently available in China, another variant developed independently by Chinese researchers is expected to enter the clinical research and trial phase in the near future [10]. A review of eight studies on the efficacy of the two vaccines found that immunisation using JYNNEOS significantly enhanced humoral immunity and induced rash, while ACAM2000 elicited a comparatively modest increase in neutralising antibodies relative to JYNNEOS, yet remained effective [11]. Another systematic review also showed that Mpox vaccines demonstrated the ability to generate adequate antibodies [12]. Effective and safe vaccination for Mpox prevention is urgent, particularly for the high risk MSM population.

Previous research has identified several factors linked to the willingness to accept Mpox vaccination. For example, concerns over vaccine efficacy, adverse effects, and safety have been shown to deter vaccination uptake [13]. In China, studies have correlated vaccination willingness with apprehensions about vaccine safety [14], knowledge of Mpox [15,16], risk perception of Mpox infection, education, and poor condom use among self-reported HIV-positive individuals [16], as well as the vaccination status of friends or sexual partners [13]. However, these studies are limited by small sample sizes, focus on the general population, or only encompass specific areas like northwestern or southwestern China, potentially limiting their generalisability. Based on the fact that China has the largest MSM population in the world, with an estimated size of 8.3 million [17], it is imperative to examine the determinants of Mpox vaccine intention within this high-risk group.

We aimed to explore the correlates of the intention of MSM to get vaccinated for free against Mpox and attempted to construct a statistical model using machine learning methods, thus providing a theoretical basis for promoting Mpox vaccination in this population.

## METHODS

### Subjects

This was a multicentre cross-sectional survey conducted between November 2023 and March 2024 in six regions of China, including Shanghai (East), Shenyang (Northeast), Guangzhou (Southeast), Xi’an (Northwest), Xinjiang (Northwest), and Kunming (Southwest). In collaboration with non-governmental organisations during the data collection phase, we recruited men aged ≥18 years old who had had sex with men and lived permanently in the survey site for the last six months. We excluded individuals who spent less than 300 seconds finishing the questionnaire, made errors in the quality control questions, and had IP addresses inconsistent with the survey site. The study guaranteed anonymity and participants had the right to withdraw at any time and without consequences. Written informed consent was obtained from all participants before the study was conducted.

### Variables and measurements

#### Vaccination intention

The main outcome of the questionnaire was the intention to receive the Mpox vaccine for free. Participants who answered ‘very willing’ and ‘willing’ were classified as having clear vaccination intention, while those who answered ‘very unwilling’, ‘unwilling’, or ‘unsure’ were classified as not having clear vaccination intention.

#### Background information

We collected the following background information: sociodemographic characteristics (*e.g.* age, education level, marital status, occupation type, monthly income, residence, homosexual orientation), behavioural characteristics (*e.g.* drinking, smoking, multiple sexual partners, unprotected anal intercourse), and disease diagnosis (*e.g.* HIV infection status, sexually transmitted diseases, history of chronic diseases, Mpox diagnosis, or symptoms).

#### Psychological characteristics

For the psychological determinants, we measured anxiety using the Chinese version of Generalized Anxiety Disorder Scale (Cronbach’s α = 0.954) [18], depression through the Chinese version of Patient Health Questionnaire (Cronbach’s α = 0.943) [19], social support using the Chinese version of Perceived Social Support Scale (Cronbach’s α = 0.958) [20], and discrimination via the Chinese version of MSM-related Discrimination Scale (Cronbach’s α = 0.873) [21].

#### Mpox-related variables

We developed a series of scales related to Mpox. The first of these scales – The Mpox knowledge scale – comprised 12 statements, which required participants to ascertain the accuracy of each statement. In terms of validity, it had a Cronbach’s α of 0.795 (Table S1 in the **Online Supplementary Document**). Participants earned 1 point for each correct response and 0 points for each incorrect response or expression of uncertainty. The aggregate score from these 12 items constituted a continuous numerical variable representing the level of Mpox knowledge [22,23].

Judgement on Mpox infection, Mpox risk perception, Mpox prevention self-efficacy [24], individual and public attitudes toward Mpox [25,26], concerns about receiving Mpox health care [27,28], and stigma when receiving Mpox health care [29,30] were all designed as 5-point Likert scales. The response options for each item were ‘strongly disagree’, ‘disagree’, ‘unsure’, ‘agree’ and ‘strongly agree’, corresponding to scores of 1–5.

#### Attitudes toward Mpox vaccination

Several questions on attitudes toward Mpox vaccination were designed based on the Protective Motivation Theory [31,32]. We asked participants to indicate their agreement or disagreement with these statements:

− Internal rewards (recognition of factors within the individual that hinder one’s willingness to be vaccinated): ‘I think I am very healthy, so there is no need to be vaccinated against Mpox.’
− External rewards (recognition of external environmental factors that hinder one’s willingness to be vaccinated): ‘There is no need for me to be vaccinated against Mpox because no one around me is vaccinated and they do not have related diseases.’
− Response efficacy (recognition of the benefits of vaccination): ‘If received the Mpox vaccine, Mpox infection can be prevented effectively.’
− Response cost (subjective and objective obstacles faced in the decision-making and behavioural process of vaccination): ‘I am worried that I will be infected with Mpox if I receive Mpox vaccines.’

### Statistical analysis

First, we split the participants randomly into a training set and a testing set in a ratio of 3:1, after which we performed descriptive statistical analysis, the Mann-Whitney U test, and the χ^2^ test. We then used the least absolute shrinkage and selection operator (LASSO) regression analysis to screen the 31 independent variables on the training set. We used the area under the curve (AUC), as one of the measures of LASSO to specify the target covariates that were minimised when cross-validating the selected model and the 1-standard error to obtain a model with excellent performance and a minimum number of independent variables. After features with nonzero coefficients in the LASSO regression analysis were selected, we performed a multivariate logistic regression analysis on the training set to construct the model, presenting our results as a nomogram. Based on the training set and the testing set, we used the receiver operating characteristic (ROC) curve, calibration curve, Kolmogorov-Smirnov (K-S) test, lift test, and population stability index (PSI) to verify the validity and stability of the model. *P*-values below 0.05 indicated statistical significance.

We conducted all analyses in *R*, version 4.3.1 (R Core Team, Vienna, Austria).

## RESULTS

### Demographic Characteristics of Participants

**Figure 1:**
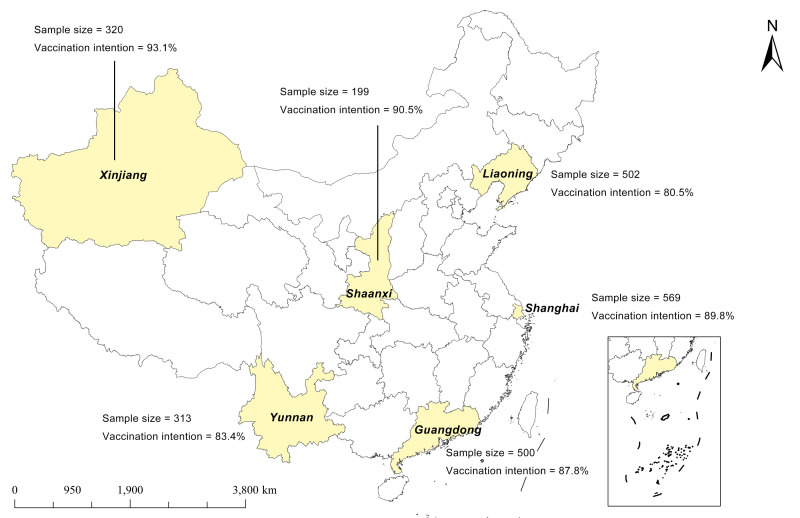
Sample size and the vaccination intention in six survey sites.

We collected 2481 questionnaires, of which 2403 (96.86%) were valid. The median age of the participants was 29 (interquartile range (IQR) = 25–35) years old. Overall, 2093 (87.1%) participants intended to receive the free Mpox vaccination, while 310 (12.9%) refused or were unsure (**Figure 1** and Table S2 in the **Online Supplementary Document**). For external validation, we divided the total sample into a training set (n = 1802 participants) and a testing set (n = 601) at a ratio of approximately 3:1. The training set contained 1571 (87.2%) participants willing to get vaccinated, with a median age of 29 (IQR = 25–35) years, while the testing test included 522 (86.9%) participants having the vaccination intention, with a median age of 29 (IQR = 25–34) years old (**Table 1**).

**Table 1:** Characteristics of the participants in different groups*

	Total (n = 2403)	Training set (n = 1802)	Testing set (n = 601)	*P*-value
**Age in years, MD (IQR)**	29 (25–35)	29 (25–35)	29 (25–34)	0.962
**Education level**				0.671
Middle school and below	515 (21.4)	382 (21.2)	133 (22.1)	
High school and above	1888 (78.6)	1420 (78.8)	468 (77.9)	
**Marital status**				0.381
Unmarried, divorced, or widowed	2148 (89.4)	531 (88.4)	1617 (89.7)	
Married	255 (10.6)	70 (11.6)	185 (10.3)	
**Occupation type**				0.516
Mental labour	386 (16.1)	285 (15.8)	101 (16.8)	
Manual labour	774 (32.2)	569 (31.6)	205 (34.1)	
Student	320 (13.3)	243 (13.5)	77 (12.8)	
Freelance	923 (38.4)	705 (39.1)	218 (36.3)	
**Monthly income in CNY**				0.732
≤3000	502 (20.9)	383 (21.3)	119 (19.8)	
3001–6000	851 (35.4)	640 (35.5)	211 (35.1)	
6001–12000	802 (33.4)	591 (32.8)	211 (35.1)	
≥12000	248 (10.3)	188 (10.4)	60 (10.0)	
**Residence**				0.787
Local	936 (39.0)	695 (38.6)	241 (40.1)	
Short term (<5 years)	870 (36.2)	658 (36.5)	212 (35.3)	
Long term (>5 years)	597 (24.8)	449 (24.9)	148 (24.6)	
**Homosexual orientation**				0.185
No	516 (21.5)	399 (22.1)	117 (19.5)	
Yes	1887 (78.5)	1403 (77.9)	484 (80.5)	
**Smoking**				0.521
No	1464 (60.9)	1105 (61.3)	359 (59.7)	
Yes	939 (39.1)	697 (38.7)	242 (40.3)	
**Drinking**				0.991
No	1122 (46.7)	842 (46.7)	280 (46.6)	
Yes	1281 (53.3)	960 (53.3)	321 (53.4)	
**Multiple sexual partners**				0.715
No	1206 (50.2)	900 (49.9)	306 (50.9)	
Yes	1197 (49.8)	902 (50.1)	295 (49.1)	
**Unprotected anal intercourse**				0.194
No	1395 (58.1)	1032 (57.3)	363 (60.4)	
Yes	1008 (41.9)	770 (42.7)	238 (39.6)	
**HIV status**				0.641
Negative or unknown	2204 (91.7)	1656 (91.9)	548 (91.2)	
Positive	199 (8.3)	146 (8.1)	53 (8.8)	
**Sexually transmitted diseases**				0.180
No	2182 (90.8)	1645 (91.3)	537 (89.4)	
Yes	221 (9.2)	157 (8.7)	64 (10.6)	
**History of chronic diseases**				0.226
No	2078 (86.5)	1549 (86.0)	529 (88.0)	
Yes	325 (13.5)	253 (14.0)	72 (12.0)	
**Mpox diagnosis/symptoms**				0.319
No	2261 (94.1)	1701 (94.4)	560 (93.2)	
Yes	142 (5.9)	101 (5.6)	41 (6.8)	
**Anxiety, MD (IQR)**	10 (7–14)	10 (7–14)	10 (7–14)	0.088
**Depression, MD (IQR)**	14 (10–18)	14 (10–18)	13 (10–18)	0.212
**Social support, MD (IQR)**	59 (48–70)	59 (48–70)	58 (48–69)	0.862
**Discrimination, MD (IQR)**	1 (0–3)	1 (0–3)	1 (0–3)	0.379
**Mpox knowledge, MD (IQR)**	8 (7–10)	8 (7–10)	8 (7–10)	0.982
**Mpox infection judgement, MD (IQR)**	17 (16–20)	16 (15–20)	17 (16–20)	0.116
**Mpox risk perception, MD (IQR)**	6 (4–6)	6 (4–6)	6 (4–6)	0.949
**Mpox prevention self-efficacy, MD (IQR)**	23 (20–25)	23 (20–25)	23 (20–25)	0.743
**Individual attitudes on Mpox, MD (IQR)**	15 (12–16)	15 (12–16)	14 (12–16)	0.269
**Public attitudes on Mpox, MD (IQR)**	15 (11–16)	15 (11–16)	15 (10–16)	0.260
**Mpox health care concern, MD (IQR)**	16 (12–20)	16 (12–20)	16 (12–20)	0.831
**Mpox health care stigma, MD (IQR)**	35 (29–39)	35 (29–39)	35 (28–39)	0.565
**Vaccination internal rewards**				0.979
Agree	397 (16.5)	297 (16.5)	100 (16.6)	
Disagree	2006 (83.5)	1505 (83.5)	501 (83.4)	
**Vaccination external rewards**				0.218
Agree	405 (16.9)	314 (17.4)	91 (15.1)	
Disagree	1998 (83.1)	1488 (82.6)	510 (84.9)	
**Vaccination response efficacy**				0.931
Agree	1750 (72.8)	1311 (72.8)	439 (73.0)	
Disagree	653 (27.2)	491 (27.2)	162 (27.0)	
**Vaccination response cost**				0.403
Agree	633 (26.3)	483 (26.8)	150 (25.0)	
Disagree	1770 (73.7)	1319 (73.2)	451 (75.0)	
**Vaccination intention**				0.892
No	310 (12.9)	231 (12.8)	79 (13.1)	
Yes	2093 (87.1)	1571 (87.2)	522 (86.9)	

*Presented as n (%) unless specified otherwise.

IQR – intequartile range, MD – median

### Model construction

**Figure 2:**
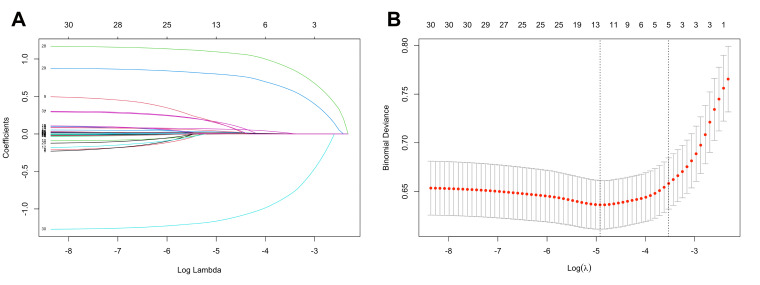
Feature selection using the LASSO regression model for Mpox vaccination intention. **Panel A.** Variable selection by the LASSO binary logistic regression model. We constructed a coefficient profile plot against the log (lambda) sequence and selected five variables with nonzero coefficients by deriving the optimal lambda. **Panel B.** Following verification of the optimal parameter (lambda) in the LASSO model, we plotted the partial likelihood deviance (AUC) curve vs the log (lambda) and drew dotted vertical lines based on the 1 standard error criteria. LASSO – least absolute shrinkage and selection operator.

**Figure 3:**
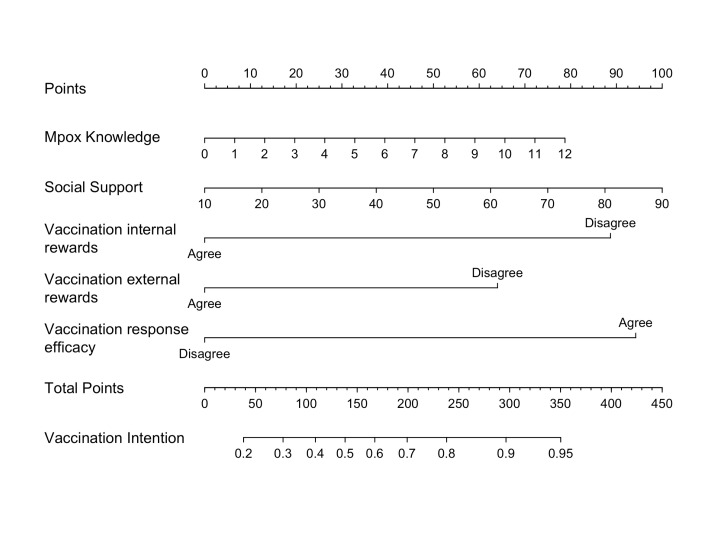
Developed nomogram for the Mpox vaccination intention. Important features of Mpox knowledge, social support, vaccination internal rewards, vaccination external rewards, and vaccination response efficacy for the nomogram model.

The features with nonzero coefficients selected by LASSO included Mpox knowledge, social support, vaccination internal rewards, vaccination external rewards and vaccination response efficacy, with lambda 1-standard error of 0.012, 0.002, 0.884, 0.596, and −0.819, respectively (**Figure 2**). We included these significant indicators in the logistic regression analysis and established their nomograms (**Figure 3**, **Table 2**). We found that Mpox knowledge (odds ratio (OR) = 1.095; 95% confidence interval (CI) = 1.036–1.157) and social support (OR = 1.018; 95% CI = 1.007–1.028) were positively associated with Mpox vaccination intention. Disagreement with statements representing internal (OR = 3.426; 95% CI = 2.182–5.379) and external (OR = 2.431; 95% CI = 1.553–3.788) barriers to vaccination significantly increased the likelihood of willingness, while disagreement with response efficacy (OR = 0.270; 95% CI = 0.197–0.370) reduced it. The nomogram visually summarises the logistic regression results, providing a practical tool to predict individual Mpox vaccination intention based on the analysed predictors.

**Table 2:** Logistic regression analysis of the predictors for Mpox vaccination intention

	β coefficient	OR (95% CI)	*P*-value
**Mpox knowledge**	0.091	1.095 (1.036–1.157)	0.001
**Social support**	0.017	1.018 (1.007–1.028)	<0.001
**Vaccination internal rewards**	1.231	3.426 (2.182–5.379)	<0.001
**Vaccination external rewards**	0.888	2.431 (1.553–3.788)	<0.001
**Vaccination response efficacy**	−1.308	0.270 (0.197–0.370)	<0.001

CI – confidence interval, OR – odds ratio

### Model validation

**Figure 4:**
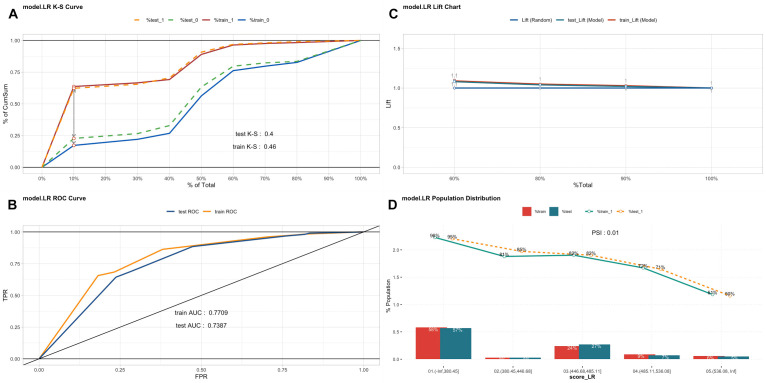
Model validation of the training set and testing set. **Panel A.** The K-S values of the model in the training set and the testing set. The red line represents the positive performance of the model in the training set, while the blue line represents the negative performance of the model in the training set. The orange line represents the positive performance of the model in the testing set, while the green line represents the negative performance of the model in the testing set. **Panel B.** The pooled AUC of the ROC curve in the training set and testing set. The y-axis represents the true positive rate of the prediction, and the x-axis represents the false positive rate of the prediction. The yellow line represents the performance of the model in the training set, and the blue line represents the performance of the model in the testing set. **Panel C.** The lift chart of the model in the training set and testing set respectively. The blue line is random, the red line represents the performance of the model in the training set, and the green line represents the performance of the model in the testing set. **Panel D.** The PSI of the model in the training set and testing set respectively. The green line represents the performance of the model in the testing set, while the orange line represents the performance of the model in the training set.

**Figure 5:**
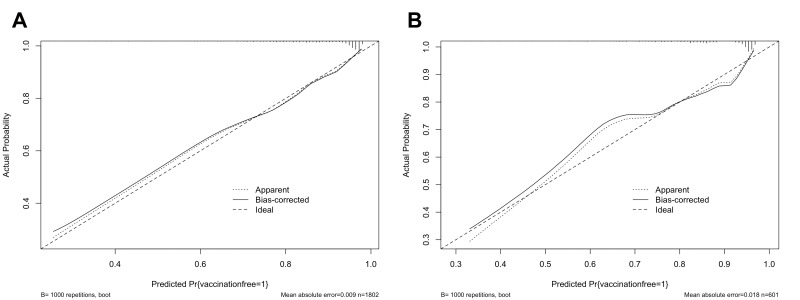
Calibration curves of the Mpox vaccination intention model. The y-axis represents actual probability of Mpox vaccination intention, the x-axis represents the predicted probability of Mpox vaccination intention. The diagonal dotted line represents a perfect prediction by an ideal model, the solid line represents the performance of each set, with the results indicating that a closer fit to the diagonal dotted line represents a better prediction. **Panel A**. Training set. **Panel B.** Testing set.

The K-S value was 0.46 for the training set and 0.40 for the testing set (**Figure 4**, Panel A). The validation results were all greater than 0.2, indicating that the risk differentiation ability of the model established in this study was strong. We used the ROC curve to evaluate the discriminatory capacity of the model (**Figure 4**, Panel B). In the ROC plot, the AUC value of the model was 0.7709 in the training set and 0.7387 in the testing set, indicating moderately good model performance. From the results of the Lift test (**Figure 4**, Panel C), we see that the values of the training set and testing set were above 1, proving that the model had a moderate predictive power. The PSI value of 0.01 was less than 0.1, indicating that the model was highly stable (**Figure 4**, Panel D). Lastly, we used calibration plots to calibrate the model. The calibration curves indicate that the model and the testing set showed a good degree of fit (**Figure 5**).

## DISCUSSION

The Mpox vaccination intention rate among the Chinese MSM population in our study was 87.1%, which follows the findings of other research (81.5–86.0%) [33–35]. A systematic review comprising 29 cross-sectional studies with 52 658 participants showed that the pooled prevalence of intention to vaccinate against Mpox was 61% [36]. The vaccination intention among the MSM population in our study and in the above-mentioned research [33–35] is higher likely than that of the general population observed in this systematic review [36] due to the relatively higher risk of Mpox infection associated with MSM. This also underscores the importance and necessity of prioritising the promotion of Mpox vaccination among susceptible groups.

We observed regional disparities in Mpox vaccination intention (Table S2 in the **Online Supplementary Document**), whereby participants from Shenyang (Northeast) and Kunming (Southwest) had notably lower vaccination intention compared to those in Shanghai (East), potentially due to a combination of geographical, economic, health care, and sociocultural factors. Higher economic development and urbanisation, coupled with more supportive social norms, appear to enhance vaccination intention, in general [37], which is also seemingly impacted by the different accessibility of vaccines and health care services across regions [38]. The proportion of ethnic minorities in a given population may also correlate with vaccine hesitancy rates [39]. Moreover, there exists a significant potential for heterogeneity within the MSM community. For instance, disparities have been found between MSM individuals residing in rural settings and their urban counterparts with respect to risk behaviours [40]. One reason might be that the former navigate multiple, intersecting identities, negotiating their roles within communities and families [41].

We employed the LASSO machine learning algorithm to construct a model for the Mpox vaccination intention. According to the results, Mpox knowledge, social support, vaccination internal rewards, vaccination external rewards, and vaccination response efficacy were significant correlates of the Mpox vaccination intention. The findings of a study on HPV vaccination intentions and behaviours among MSM individuals that used structural equation modelling concur with ours regarding the importance of disease and virus-related knowledge, as it concluded that HPV knowledge significantly predicts vaccination intention (*P* = 0.02) [42]. Another study on COVID-19 vaccination willingness among the mobile population also used machine learning for variable selection and similarly emphasised the significance of perceived social support [43]. The model performance in their study (training set: AUC = 0.8076, K-S value = 0.47) was slightly superior to ours (training set: AUC = 0.7709, K-S value = 0.46), potentially due to the distinct characteristics of our study population (*i.e.* MSM).

Consistent with the findings of several studies, we found that an increase in knowledge about Mpox is closely associated with higher intent to get vaccinated [15,16,33,44]. One reasonable assumption for this is that individuals with a more comprehensive grasp of knowledge can better understand the consequences of Mpox and the correct methods for its prevention and control, making them more inclined to get vaccinated. This also highlights the significance of conducting health education initiatives targeting the MSM population. Socioeconomic status may influence knowledge levels, which in turn could affect vaccination intention. As an example, one study indicated that individuals with lower socioeconomic status (*e.g.* lower income, residing in smaller towns) may be more susceptible to vaccine-related misinformation, thereby impacting their views on vaccination benefits and potentially reducing their willingness to get vaccinated [45]. This study concluded that social support is a protective factor for vaccination intention. Other research on the Dutch MSM population found a positive correlation between the lack of connection with the gay/queer community and the reluctance to get vaccinated [33]. Mpox is an emerging infectious disease, and the specific vaccination is also a newly introduced medical procedure not yet widely adopted in China. If individuals do not perceive support from friends, family, or their community, and have no outlet for their doubts or concerns, we hypothesise that this may lead to the vaccination hesitancy.

According to the Protection Motivation Theory [31,32], internal rewards refer to factors that promote an individual to engage in risky behaviour, or the benefits derived from adopting such risky behaviour. Our findings suggest that a higher perception of internal rewards leads to a lower intention to get vaccinated. An Mpox survey among people experiencing homelessness in San Francisco also corroborates this point; among those who refused vaccination, 30% stated that they did not need it because they perceived themselves as not being at risk [46]. When individuals strongly identify with these factors that hinder vaccination, their willingness to be vaccinated decreases.

External rewards from the Protection Motivation Theory refer to factors within the external environment, such as peers or family, that reinforce an individual’s risky behaviour. Consistent with previous findings [13], MSM individuals in our study were more likely to get vaccinated if their friends or sexual partners have been vaccinated, and vice versa. The behaviours and comments of people around can influence one’s judgments. Among MSM, peers and social networks, particularly influential advocators within the community, exert a significant impact. They are pivotal in bridging health care information gaps, fostering trust, and alleviating stigma among MSM members. Peer-driven social network interventions, serving as external rewards, are thus instrumental in addressing vaccine hesitancy related to stigma and distrust [47].

Response efficacy refers to an individual’s cognition of whether engaging in health behaviours will result in benefits [31,32]. Numerous studies have reflected the association between high response efficacy and high vaccination intention. For instance, in the aforementioned study among the homeless, 88% of the respondents agreed that vaccinating against Mpox effectively protects health [46]. In a survey of university students in southwest China, 70% of the participants had confidence in the vaccine [44]. Nevertheless, negative attitudes towards vaccines can be nuanced and can include concerns over side effects, partial protection, and efficacy against viral variants. These factors may contribute to a moderate decline in vaccine response efficacy, which may lead to a decrease in vaccination intention [48,49]. This suggests that public health campaigns could more effectively promote vaccines by addressing these specific concerns [50].

Our results also present a profile of the MSM population from a public health perspective, which suggests that, when an effective and favourable Mpox vaccine becomes available in the future, individuals with such characteristics may be more inclined to get vaccinated. Our findings provide reference for designing and formulating public health strategies, thereby enhancing the promotion of Mpox vaccination. Feasible intervention measures include, for instance, disseminating information through media outlets to improve the public’s comprehension and confidence in vaccine protocols, as well as leveraging non-governmental organisations and LGBTQ+ organisations to facilitate peer-led and community-focused educational programs. Furthermore, within the sociocultural context of China, it is essential to curtail the undue emphasis on sexual minority groups and to instead promote the vaccines by adopting a health-centric perspective.

There are several limitations to our study. First, it is limited to a cross-sectional survey that captured participants’ self-reported vaccination intentions. Future longitudinal studies are essential to determine whether these intentions translate into actual vaccination behaviour. Second, the sensitive nature of topics in MSM studies may elicit social desirability bias. To address this, we guaranteed participants’ anonymity and partnered with non-governmental organisations to enhance participants’ trust. Third, the reliance on self-reported data may introduce recall bias. Fourth, considering the potential heterogeneity within the MSM population, we recommend that future research collect additional data and increase the sample size to improve generalisability.

## CONCLUSIONS

Mpox knowledge, social support, vaccination internal rewards, vaccination external rewards, and vaccination response efficacy are meaningful correlates and potential predictors for Mpox vaccination. Thus, health education on Mpox as well as its vaccination is necessary. The government should take the lead in formulating targeted public health strategies through a whole-of-society approach, making full use of peer and community education to promote and popularise Mpox vaccination.

## Additional material


Online Supplementary Document

## References

[1] Harris E What to Know About Monkeypox. JAMA. 2022;327:2278–9. 10.1001/jama.2022.9499 35622356

[2] Ladnyj ID Ziegler P Kima E A human infection caused by monkeypox virus in Basankusu Territory, Democratic Republic of the Congo. Bull World Health Organ. 1972;46:593–7. 4340218 PMC2480792

[3] Parker S Buller RM A review of experimental and natural infections of animals with monkeypox virus between 1958 and 2012. Future Virol. 2013;8:129–57. 10.2217/fvl.12.130 23626656 PMC3635111

[4] Gessain A Nakoune E Yazdanpanah Y Monkeypox. N Engl J Med. 2022;387:1783–93. 10.1056/NEJMra2208860 36286263

[5] World Health Organization. Multi-country outbreak of mpox, External situation report#32- 30 April 2024. Geneva, Switzerland: World Health Organization; 2024. Available: https://www.who.int/publications/m/item/multi-country-outbreak-of-mpox–external-situation-report-32–30-april-2024. Accessed: 27 February 2025.

[6] World Health Organization. WHO Director-General declares mpox outbreak a public health emergency of international concern. 14 August 2024. Available: https://www.who.int/news/item/14-08-2024-who-director-general-declares-mpox-outbreak-a-public-health-emergency-of-international-concern. Accessed: 27 February 2025.

[7] Antinori A Mazzotta V Vita S Carletti F Tacconi D Lapini LE Epidemiological, clinical and virological characteristics of four cases of monkeypox support transmission through sexual contact, Italy, May 2022. Euro Surveill. 2022;27:2200421. 10.2807/1560-7917.ES.2022.27.22.2200421 35656836 PMC9164671

[8] Perez Duque M Ribeiro S Martins JV Casaca P Leite PP Tavares M Ongoing monkeypox virus outbreak, Portugal, 29 April to 23 May 2022. Euro Surveill. 2022;27:2200424. 10.2807/1560-7917.ES.2022.27.22.2200424 35656830 PMC9164676

[9] Minhaj FS Rao AK McCollum AM Imported Monkeypox from International Traveler, Maryland, USA, 2021. Emerg Infect Dis. 2022;28:1738. 10.3201/eid2808.220726 35798002 PMC9328915

[10] Zhao R Wu L Sun J Liu D Han P Gao Y Two noncompeting human neutralizing antibodies targeting MPXV B6 show protective effects against orthopoxvirus infections. Nat Commun. 2024;15:4660. 10.1038/s41467-024-48312-2 38821921 PMC11143242

[11] Kandeel M Morsy MA Abd El-Lateef HM Marzok M El-Beltagi HS Al Khodair KM Efficacy of the modified vaccinia Ankara virus vaccine and the replication-competent vaccine ACAM2000 in monkeypox prevention. Int Immunopharmacol. 2023;119:110206. 10.1016/j.intimp.2023.110206 37087871 PMC10120163

[12] Ghazy RM Elrewany E Gebreal A ElMakhzangy R Fadl N Elbanna EH Systematic Review on the Efficacy, Effectiveness, Safety, and Immunogenicity of Monkeypox Vaccine. Vaccines (Basel). 2023;11:1708. 10.3390/vaccines11111708 38006040 PMC10674429

[13] Huang X Lin Z Qin J Yu D Zhang F Fang G Willingness to accept monkeypox vaccine and its correlates among men who have sex with men in Southern China: a web-based online cross-sectional study. Front Public Health. 2024;12:1289918. 10.3389/fpubh.2024.1289918 38384873 PMC10879393

[14] Li LL Han MJ Lyu P Tang HL Yang J Zhang W [Survey on monkeypox knowledge awareness, risk perception and vaccination intention in men who have sex with men in five cities in northeast China]. Zhonghua Liu Xing Bing Xue Za Zhi. 2024;45:128–33. Chinese. 38228535 10.3760/cma.j.cn112338-20230728-00047

[15] Dong C Yu Z Zhao Y Ma X Knowledge and vaccination intention of monkeypox in China’s general population: A cross-sectional online survey. Travel Med Infect Dis. 2023;52:102533. 10.1016/j.tmaid.2022.102533 36543284 PMC9759477

[16] Zheng M Qin C Qian X Yao Y Liu J Yuan Z Knowledge and vaccination acceptance toward the human monkeypox among men who have sex with men in China. Front Public Health. 2022;10:997637. 10.3389/fpubh.2022.997637 36388271 PMC9640956

[17] Hu M Xu C Wang J Spatiotemporal Analysis of Men Who Have Sex With Men in Mainland China: Social App Capture-Recapture Method. JMIR Mhealth Uhealth. 2020;8:e14800. 10.2196/14800 32012086 PMC7007599

[18] Sun J Liang K Chi X Chen S Psychometric Properties of the Generalized Anxiety Disorder Scale-7 Item (GAD-7) in a Large Sample of Chinese Adolescents. Healthcare (Basel). 2021;9:1709. 10.3390/healthcare9121709 34946435 PMC8701121

[19] Wang W Bian Q Zhao Y Li X Wang W Du J Reliability and validity of the Chinese version of the Patient Health Questionnaire (PHQ-9) in the general population. Gen Hosp Psychiatry. 2014;36:539–44. 10.1016/j.genhosppsych.2014.05.021 25023953

[20] Chou KL Assessing Chinese adolescents’ social support: the multidimensional scale of perceived social support. Pers Individ Dif. 2000;28:299–307. 10.1016/S0191-8869(99)00098-7

[21] Denney MR Pichon LC Brantley ML Violence, Discrimination, Psychological Distress, and HIV Vulnerability Among Men Who Have Sex With Men in Memphis, Tennessee. Am J Mens Health. 2023;17:15579883231163727. 10.1177/15579883231163727 36992529 PMC10064477

[22] Centers for Disease Control and PreventionMpox. Available: https://www.cdc.gov/poxvirus/mpox/index.html. Accessed.

[23] Chinese Center for Disease Control and Prevention. Mpox prevention and control propaganda core information. 2023. Available: https://www.chinacdc.cn/jkzt/crb/qt/szkb_13037/kpzl/202308/t20230802_268336.html. Accessed.

[24] Winters M Malik AA Omer SB Attitudes towards Monkeypox vaccination and predictors of vaccination intentions among the US general public. PLoS One. 2022;17:e0278622. 10.1371/journal.pone.0278622 36454991 PMC9714903

[25] Baugher AR Beer L Fagan JL Mattson CL Freedman M Skarbinski J Prevalence of Internalized HIV-Related Stigma Among HIV-Infected Adults in Care, United States, 2011-2013. AIDS Behav. 2017;21:2600–8. 10.1007/s10461-017-1712-y 28213821 PMC5555833

[26] Luz PM Torres TS Almeida-Brasil CC Marins LMS Bezerra DRB Veloso VG Translation and validation of the Short HIV Stigma scale in Brazilian Portuguese. Health Qual Life Outcomes. 2020;18:322. 10.1186/s12955-020-01571-1 33008400 PMC7530962

[27] Gillman AS Scharnetzki L Boyd P Ferrer RA Klein WMP Han PKJ Perceptions and tolerance of uncertainty: relationship to trust in COVID-19 health information and vaccine hesitancy. J Behav Med. 2023;46:40–53. 10.1007/s10865-022-00302-9 35394240 PMC8990605

[28] Lyu X Liu Y Yu H Mi M Shang L Zhong Y Development and validation of a risk perception scale of medical help-seeking behavior in Chinese adults. Ann Transl Med. 2020;8:1352. 10.21037/atm-20-1656 33313097 PMC7723601

[29] Kupferschmidt K Why monkeypox is mostly hitting men who have sex with men. Science. 2022;376:1364–5. 10.1126/science.add5966 35737802

[30] Wagner AC Hart TA McShane KE Margolese S Girard TA Health care provider attitudes and beliefs about people living with HIV: Initial validation of the Health Care Provider HIV/AIDS Stigma Scale (HPASS). AIDS Behav. 2014;18:2397–408. 10.1007/s10461-014-0834-8 24965675

[31] Maddux JE Rogers RW Protection motivation and self-efficacy: A revised theory of fear appeals and attitude change. J Exp Soc Psychol. 1983;19:469-79. 10.1016/0022-1031(83)90023-9

[32] Prentice-Dunn S Rogers RW Protection Motivation Theory and preventive health: beyond the Health Belief Model. Health Educ Res. 1986;1:153-61. 10.1093/her/1.3.153

[33] Dukers-Muijrers NH Evers Y Widdershoven V Davidovich U Adam PCG Op de Coul ELM Mpox vaccination willingness, determinants, and communication needs in gay, bisexual, and other men who have sex with men, in the context of limited vaccine availability in the Netherlands (Dutch Mpox-survey). Front Public Health. 2023;10:1058807. 10.3389/fpubh.2022.1058807 36684959 PMC9850232

[34] Paparini S Whitacre R Smuk M Thornhill J Mwendera C Strachan S Public understanding and awareness of and response to monkeypox virus outbreak: A cross-sectional survey of the most affected communities in the United Kingdom during the 2022 public health emergency. HIV Med. 2023;24:544–57. 10.1111/hiv.13430 36385726

[35] Gilbert M Ablona A Chang HJ Grennan T Irvine MA Sarai Racey C Uptake of Mpox vaccination among transgender people and gay, bisexual and other men who have sex with men among sexually-transmitted infection clinic clients in Vancouver, British Columbia. Vaccine. 2023;41:2485–94. 10.1016/j.vaccine.2023.02.075 36894397 PMC9990897

[36] León-Figueroa DA Barboza JJ Valladares-Garrido MJ Sah R Rodriguez-Morales AJ Prevalence of intentions to receive monkeypox vaccine. A systematic review and meta-analysis. BMC Public Health. 2024;24:35. 10.1186/s12889-023-17473-y 38166776 PMC10763398

[37] Huang Q Abad N Bonner KE Baack B Petrin R Hendrich MA Explaining demographic differences in COVID-19 vaccination stage in the United States - April-May 2021. Prev Med. 2023;166:107341. 10.1016/j.ypmed.2022.107341 36372280 PMC9650505

[38] Nguyen KH Nguyen K Corlin L Allen JD Chung M Changes in COVID-19 vaccination receipt and intention to vaccinate by socioeconomic characteristics and geographic area, United States, January 6 - March 29, 2021. Ann Med. 2021;53:1419–28. 10.1080/07853890.2021.1957998 34482788 PMC8425688

[39] Morales DX Paat YF Hesitancy or Resistance? Differential Changes in COVID-19 Vaccination Intention Between Black and White Americans. J Racial Ethn Health Disparities. 2024;11:23-35. 10.1007/s40615-022-01494-1 36547772 PMC9774084

[40] Chen W Chen L He L Chai C Urban-rural disparity in risky sexual behavior, HIV knowledge, and healthy practices among men who have sex with men: A cross-sectional study in Southeast China. PLoS One. 2024;19:e0312006. 10.1371/journal.pone.0312006 39527536 PMC11554129

[41] Quinn K Dickson-Gomez J Homonegativity, Religiosity, and the Intersecting Identities of Young Black Men Who Have Sex with Men. AIDS Behav. 2016;20:51–64. 10.1007/s10461-015-1200-1 26373283 PMC4718745

[42] Yao PY Lin CY Ko NY Zou H Lee CW Strong C Predicting human papillomavirus vaccine uptake in men who have sex with men the influence of vaccine price and receiving an HPV diagnosis. BMC Public Health. 2022;22:28. 10.1186/s12889-021-12396-y 34991553 PMC8740414

[43] Hu F Gong R Chen Y Zhang J Hu T Chen Y Prediction Model for COVID-19 Vaccination Intention among the Mobile Population in China: Validation and Stability. Vaccines (Basel). 2021;9:1221. 10.3390/vaccines9111221 34835154 PMC8617731

[44] Yang X Yang X Jiang W Luo N Hu Y Yang Y A cross-sectional investigation of factors influencing mpox vaccine hesitancy for students in Southwest China. Hum Vaccin Immunother. 2024;20:2309704. 10.1080/21645515.2024.2309704 38300140 PMC10841021

[45] Lu J Xiao Y Do Socioeconomic Disparities Matter? Unraveling the Impacts of Online Vaccine Misinformation on Vaccination Intention During the COVID-19 Pandemic in China. J Health Commun. 2023;28:91–101. 10.1080/10810730.2023.2185320 36855812

[46] Filardo TD Prasad N Waddell CJ Persad N Pellegrini GJ Jr Borne D Mpox vaccine acceptability among people experiencing homelessness in San Francisco - October-November 2022. Vaccine. 2023;41:5673–7. 10.1016/j.vaccine.2023.07.068 37591706

[47] Quinn KG Christenson E Spector A Amirkhanian Y Kelly JA The Influence of Peers on PrEP Perceptions and Use Among Young Black Gay, Bisexual, and Other Men Who Have Sex with Men: A Qualitative Examination. Arch Sex Behav. 2020;49:2129–43. 10.1007/s10508-019-01593-x 32016815 PMC7321862

[48] Hasanzad M Namazi H Larijani B COVID-19 anti-vaccine attitude and hesitancy. J Diabetes Metab Disord. 2022;22:1–4. 10.1007/s40200-022-01018-y 36373157 PMC9638374

[49] Mamani-Benito O Farfán-Solís R Huayta-Meza M Tito-Betancur M Morales-García WC Tarqui EEA Effect of religious fatalism and concern about new variants on the acceptance of COVID-19 vaccines. Front Psychiatry. 2023;14:1071543. 10.3389/fpsyt.2023.1071543 36937730 PMC10017722

[50] Jensen UT Ayers S Koskan AM Video-based messages to reduce COVID-19 vaccine hesitancy and nudge vaccination intentions. PLoS One. 2022;17:e0265736. 10.1371/journal.pone.0265736 35385505 PMC8985948

