# Supplementary material for: Psychosocial correlates of free Mpox vaccination intention among men who have sex with men in China: model construction and validation
Source: J Glob Health. 2025 Apr 2;15:04070. doi: 10.7189/jogh.15.04070 (PMC11961056; doi:10.7189/jogh.15.04070)
Supplement: Online Supplementary Document [file jogh-15-04070-s001.pdf]

Supplement to: Zhang Y, Zhang M, Wang Y, Li C, Xu H, Xu G, Zhang J, Wang Y, Hu F, Cai Y. Psychosocial correlates of free Mpox vaccination intention among men who have sex with men in China: model construction and validation. J Glob Health. 2025;15:04070.

Supplementary Materials

1. Mpox knowledge scale and its scoring method

Mpox knowledge scale contained 12 statements asking participants to determine whether it is right or wrong (Table S1). These statements primarily addressed the infectious agent of Mpox, modes of transmission, and susceptible populations. Correctly framed statements included items 1, 5, 6, 10, 11, and 12, with the remaining items being inaccurately phrased. Participants earned 1 point for each correct response and 0 points for each incorrect response or expression of uncertainty. The aggregate score from these 12 items constituted a continuous numerical variable representing the level of Mpox knowledge.

Table S1. Mpox knowledge scale

Mpox knowledge related statements	Right	Wrong	Unsure
1. Mpox is a viral disease.	1	2	3
2. Mpox does not kill people.	1	2	3
3. Mpox only affects men.	1	2	3
4. Antibiotics are effective against mpox infections.	1	2	3
5. The source of mpox infection is either an infected animal or an infected person.	1	2	3
6. Mpox can be transmitted through mucous membranes and broken skin.	1	2	3
7. One cannot get mpox from hugging or kissing.	1	2	3
8. One cannot get mpox from oral sex or touching an infected person's genitals.	1	2	3
9. The only sign of mpox is a rash.	1	2	3
10. People infected with mpox usually have no symptoms during the incubation period.	1	2	3
11. The correct use of condoms can reduce the chance of contracting mpox.	1	2	3
12. Vaccination against smallpox can be used to prevent mpox.	1	2	3

2. The regional disparities of Mpox vaccination intention

Table S2 presents the number of participants across the six survey sites, the frequency distribution of Mpox vaccination intention among the MSM population, and the results of univariate logistic regression with Shanghai as the reference group. Compared to Shanghai, the intention to receive the Mpox vaccine among MSM in Shenyang and Kunming was significantly lower, with odds ratios of 0.47 (95% CI: 0.33, 0.66) and 0.57 (95% CI: 0.38, 0.85), respectively.

Table S2. Distribution and univariate logistic regression of different locations

Location	Overall (N=2,403)	Vaccination Intention		OR (95%CI)
		No (N=310)	Yes (N=2,093)	
Shanghai	569 (23.7)	58 (18.7)	511 (24.4)	Ref
Guangzhou	500 (20.8)	61 (19.7)	439 (21.0)	0.82 (0.56, 1.20)
Shenyang	502 (20.9)	98 (31.6)	404 (19.3)	0.47 (0.33, 0.66) ***
Xi'an	199 (8.3)	19 (6.1)	180 (8.6)	1.08 (0.63, 1.90)
Kunming	313 (13.0)	52 (16.8)	261 (12.5)	0.57 (0.38, 0.85) **
Xinjiang	320 (13.3)	22 (7.1)	298 (14.2)	1.54 (0.94, 2.61)

Notes: categorical variables are expressed as frequency (percentage); statistical significance marked as ***P<0.001, **P<0.01
